# Use of a conditional Glanzmann thrombasthenia mouse model reveals a supportive and possibly non-adhesive role for TLT-1 in the platelet-fibrinogen interaction

**DOI:** 10.1080/09537104.2025.2574379

**Published:** 2025-11-07

**Authors:** Siobhan Branfield, Nicholas Koshy, Yashieta Somani, Barbara Manfredi, Caitlin Schneider, Hanan Tlais, Vandre Figueiredo, Randal Westrick, A. Valance Washington

**Affiliations:** a Department of Biological Sciences, Oakland University, Rochester, MI, USA; b Department of Bioengineering, Institute for Data Science, Oakland University, Rochester, MI, USA; c Life Sciences Institute, University of Michigan, Ann Arbor, MI, USA

**Keywords:** Fibrinogen, hemostasis, thrombosis, platelets, TLT-1, αIIbβ3

## Abstract

The platelet integrin receptor, αIIbβ3, binds fibrinogen to mediate platelet-platelet contacts, regulate hemostasis, and modulate inflammation. The Triggering Receptor Expressed in Myeloid (TREM) – Like (TLT)-1 is an enigmatic 34kD receptor found on platelets that affects their hemostatic and inflammatory functions. Similar to αIIbβ3, TLT-1’s ligand is also fibrinogen; however, TLT-1’s direct role in platelet function remains unknown. We created a tamoxifen-inducible conditional αIIb deficient (^*c*^*Itga2b*^*−/−*^) mouse to better understand TLT-1’s role in platelet function, specifically TLT-1’s binding to fibrinogen and its role in hemostasis and inflammation. We first characterized our ^*c*^*Itga2b*^*−/−*^ null mouse and subsequently crossed this mouse with a Treml1^−/−^ mouse, creating a conditional double knockout (^c^DKO). While the floxed ^*c*^*Itga2b /Treml1*^*−/−*^ mouse shows significant differences compared to control mice, deleting Treml1 from the ^*c*^*Itga2b*^*−/−*^ mouse results in only minor differences from the ^*c*^*Itga2b*^*−/−*^ strain in bleeding, aggregation, fibrinogen deposition and platelet spreading assays. Our data suggest that while TLT-1 plays a visible role in hemostasis, it primarily supports aggregation but may not function as an adhesive component.

## Introduction

Platelets are pivotal players in hemostasis, ensuring vascular integrity and preventing excessive bleeding. Dysfunctions in platelet activity contribute significantly to various diseases, including atherosclerosis, thrombosis, and inflammatory conditions, underscoring their critical role in maintaining health and disease pathogenesis.^1–3^ Platelet-mediated atherosclerosis and thrombosis are a leading cause of cardiovascular disease and stroke—the first and fifth leading causes of death in the United States in 2020, respectively.^1^ Platelets also play a key role in the immune response to trauma and sepsis.^4–8^ Exploring platelet function enables the advancement of targeted therapeutic interventions for diseases involving either thrombosis or inflammation.

Platelets facilitate clot formation, with one of the key steps in the process being the binding of fibrinogen to activated platelets. Fibrinogen is a glycoprotein that circulates in plasma at relatively high concentrations (~2–4 mg/mL), and its interaction with the integrin αIIbβ3 is crucial for platelet aggregation and clot formation.^9,10^ With approximately 80, 000 copies per platelet, the heterodimeric αIIbβ3 integrin receptor encoded by the *ITGA2B and ITGB3* genes serves as the major fibrinogen binding receptor on platelets. Disruptions in αIIbβ3 function lead to Glanzmann’s thrombasthenia, a rare bleeding disorder characterized by defective platelet aggregation and fibrin clot formation.^11,12^ Glanzmann’s thrombasthenia illustrates the importance of αIIbβ3 in facilitating clot formation.

At the interface of the platelet’s hemostatic and inflammatory functions stands the Triggering Receptor Expressed on Myeloid (TREM) – Like transcript (TLT)-1, an enigmatic 34kD receptor that is stored in the α-granules and binds fibrinogen.^13^ TLT-1 is encoded by the *Treml1* gene. In a recent study we demonstrated that the expression of TLT-1 in Human Embryonic Kidney 293 cells significantly enhances their adhesion to a fibrinogen matrix,^14^ supporting the hypothesis that TLT-1’s interaction with fibrinogen may be adhesive in nature. The coexistence of αIIbβ3 and TLT-1 as platelet fibrinogen receptors poses intriguing questions regarding their distinct contributions to hemostasis and inflammation. While αIIbβ3’s role in hemostasis is well established, TLT-1’s functions in these processes remain relatively unexplored, despite its significant implications in sepsis and other inflammatory conditions.^15^ The relative contributions of α_IIb_β_3_ and TLT-1 in hemostasis and inflammation could be studied via the comparison of existing mouse lines with a double-knockout (DKO) model; however, removal of these genes may hinder normal development and cause premature mortality via hemorrhage.^16^ In addition to the potential of embryonic lethality, conventional knockout of *Itgb3* results in disruption of integrin function in several lineages and has multiple secondary phenotypes and complications,^17^ making it less feasible to study a cross between the *Itgb3*^*−/−*^ and *Treml1*^*−/−*^ strains.

To gain a better understanding of TLT-1’s role in hemostasis and inflammation, we used Cre knockout technology to create a conditional knockout of αIIbβ3 by removing the *itga2b* gene, restricting the integrin loss to the platelet lineage. Our results showed that this conditional knockout reproduced the Glanzmann’s phenotype. We crossed these conditional Glanzmann’s thrombasthenia mice onto the *Treml1*^−/−^ background to investigate how these two receptors regulate platelet/fibrinogen interactions and subsequently characterized the conditional double knockout (^*c*^*DKO*) in basic hemostatic assays and evaluated fibrinogen deposition in the lungs after lipopolysaccharide (LPS) treatment.

## Materials and methods

### Antibodies

The following antibodies were used in this study: Ms CD41 PE MWReg30 (cat. Num. 558 040-BD Biosciences), Ms Ly-6 G/Ly-6C FITC RB6–8C5 (cat. Num. 553 127-BD Biosciences), Rat IgG1 Kpa It CI PE R3–34 (cat. Num. 553 925-BD Biosciences), Rat igG2b Kpa ItCI Alexa 647 A95–1 (cat. Num. 557 691-BD Biosciences), FITC Rat IgG2b (cat. Num. 553 988-BD-Biosciences), Rabbit anti TLT-1 Voldemort (produced in house and labeled with Thermo Fisher antibody labeling kit (cat number A0009), Rat anti-Ms IgG1 CD41 clone MWReg30 (cat. Num. 553 847-BD Biosciences), Rhodamine-phalloidin (cat. Num. 711–025–152-Jackson Immuno Research), 2-(4-Amidinophenyl)-6-indolecarbamidine dihydrochloride, 4′,6-Diamidino-2-phenylindole dihydrochloride (Dapi) (D9542-SIGMA Aldrich).

### Mice

All studies were approved by the Oakland University IACUC (protocol number 2021–1128) and conducted in accordance with the “Guide for the Care and Use of Laboratory Animals: Eighth Edition.” Control wildtype C57BL/6J mice, ^c^*Itga2b*^*−/−*^, *Itga2b*^*fl/fl*^*/treml1*^*+/+*^, ^c^*Itga2b*^*−/−*^*/treml1*^*−/−*^, and *Itga2b*^*fl/fl*^ mice were bred and maintained under pathogen-free conditions at Oakland University (Rochester, MI). For details of the creation of the conditional *Itga2b* mouse model see supplemental data.

### Tamoxifen administration

At 8–10 weeks of age, experimental ^c^Itga2b^fl/fl^ mice were switched to a tamoxifen-infused diet (40 mg/kg) for a 3-week period. Oral administration of tamoxifen via chow (Envigo, Indianapolis, IN) was used due to its reductions of stress and adverse effects endured by mice compared to other methods, such as intraperitoneal injection or oral gavage.^18^ The αIIbβ3 phenotype was further verified by flow cytometry of activated platelets using anti-CD41 PE. Alexa CD647-labeled Voldemort antibody was used to identify αIIbβ3 and TLT-1. Flow cytometric analyses were performed on a FACS Canto II, and the data were analyzed using FlowJo software, version 10.8.0. To control for any effects tamoxifen diet, age matched wild type and *treml1*^*−/−*^ mice were compared two weeks after a three-week regiment of tamoxifen or chow diet using basic coagulation assays. There were no significant differences with or without tamoxifen diet. The aggregation curves are shown in Supplemental Figure S4.

### Isolation of platelet rich plasma (PRP) and washed murine platelets

Blood for platelet-rich plasma (PRP) was obtained by cardiac puncture from mice anesthetized with ketamine (200 mg/kg) and xylazine (10 mg/kg), collected into syringes containing 3.2% sodium citrate. Samples were centrifuged at 100 × g for 10 minutes to remove red blood cells. Blood from 1–3 mice of the same genotype was pooled. Platelet counts were adjusted as necessary using platelet-poor plasma (PPP) from mice of the same genotype, supplemented with apyrase (0.02 U/mL) and prostaglandin E1 (PGE1, 300 nM).

For washed platelets, PRP was centrifuged at 400 × g for 5 minutes. The platelet pellet was washed in Tyrode’s buffer (134 mM NaCl, 2.9 mM KCl, 0.34 mM Na2HPO4, 1 mM MgCl2, 10 mM HEPES, 5 mM D-glucose, 0.3% bovine serum albumin, pH 7.4) containing 10% acid citrate dextrose and apyrase (0.02 U/mL) and PGE1 (300 nM). Platelets were then resuspended in Tyrode’s buffer supplemented with apyrase (0.02 U/mL) and PGE1 (300 nM).

### Platelet aggregation

Blood was taken via heart punch of anesthetized mice as described above. Mice of the same sex and strain were combined to obtain enough platelets for light aggregometry. Platelet aggregation studies were performed as described previously for washed platelet light transmission aggregometry^19^ and for whole blood impedance aggregometry we used our detailed methods published in reference.^20^

### Bleeding-time assays

The bleeding-time measurements were performed as described previously.^19^ Briefly, mice were anesthetized with intraperitoneal ketamine and xylazine (200 mg/kg and 10 mg/kg respectively) and maintained on a heating pad at 37°C. The distal 2 mm of the tail tip was transected using a sterile scalpel, and the tail was immediately immersed in 37°C isotonic saline. Bleeding was monitored visually with a stopwatch. Bleeding was considered to have ceased when no blood was observed for 60 consecutive seconds. A maximum observation time of 10 minutes was set, after which bleeding was recorded as censored.

### Platelet spreading on fibrinogen

Platelet spreading assays on immobilized fibrinogen were prepared as described previously.^21^ Blood was taken via cardiac puncture of anesthetized mice. For actin staining, 1 × 10^6^ washed platelets were allowed to bind fibrinogen coated slides for 30 min and then permeabilized with cytofix/cytoperm (BD sciences) and incubated with Rhodamine phalloidin for 45 minutes. After incubation, platelets were washed twice with Tyrode’s buffer, and slides were mounted and visualized on a Nikon C2 confocal microscope at 40x. Platelet spreading was assessed by measuring the number and area of platelets bound to immobilized fibrinogen in 7 fields of each slide using ImageJ Fiji software.

### Fibrinogen binding by flowcytometry

Washed platelets were isolated from whole blood as described above and adjusted to a concentration of 1.5 × 10^5^/μL with Tyrode’s and incubated with 10 μm FITC-Labeled fibrinogen (Innovative research catalog: IMSFBGFITC1MG), and 10 μm ADP for 15 minutes, spun down by centrifugation, washed in PBS, spun down and resuspended in FACs buffer for flow cytometric analysis of fibrinogen binding using the FACS Canto II (BD Biosciences).

### LPS-induced acute lung injury model

The LPS-induced acute lung injury model was performed as described previously.^22^

### Confocal microscopy

Confocal analyses were completed with a NIS-Elements Confocal inverted microscope equipped with 4X, 10X, 20X, 40X oil, 60X oil, 100X oil objectives. Cell area of intensity was quantified using the Nikon Instruments Software.

### Hematocrit measurement

Blood samples were taken by retro-orbital bleed using 75-mm heparinized capillaries and hematocrit measurement was performed following our detailed published guidelines described in.^23^

### Hemoglobin measurement

Blood samples were taken from each genotype by retro-orbital bleed using 75-mm heparinized capillaries and hemoglobin was measured using the Hemoglobin Assay Kit Catalog Number MAK115 (Sigma-Aldrich) and were performed as described previously.^24^ Briefly, Hemoglobin concentration was measured using the Hemoglobin Assay Kit (Sigma-Aldrich, Cat. No. MAK115) following the manufacturer’s protocol. Briefly, whole blood samples were diluted 1:250 in assay buffer, and 50 μL of each diluted sample or standard was added to a 96-well plate. Then, 50 μL of the working reagent was added to each well, mixed, and incubated at room temperature for 5 minutes. Absorbance was measured at 400 nm using a microplate reader. Hemoglobin concentrations were calculated from a standard curve generated with known concentrations of hemoglobin and expressed as g/dL. All samples were assayed in duplicate.

### Clot retraction

Blood was taken from mice via cardiac puncture. Platelet-rich plasma of mice of the same sex and strains were combined and then adjusted to 2×10^5^ plt/μL with Tyrode’s buffer (137 mM NaCl, 2.7 mM KCl, 1.8 mM CaCl_2_, 0.49 mM MgCl_2_, 11.9 mM NaHCO_3_, 0.42 mM NaH_2_PO_4_, and 5.55 mM glucose. In a glass tube, 0.4 mL of PRP was mixed with thrombin (1 U/mL) and calcium chloride (10 mM) and incubated at 37°C for 1 hour and assessed for retraction every 15 minutes. The degree of retraction was quantified by evaluating the images using image J.

### Statistics

**Figure 1.**
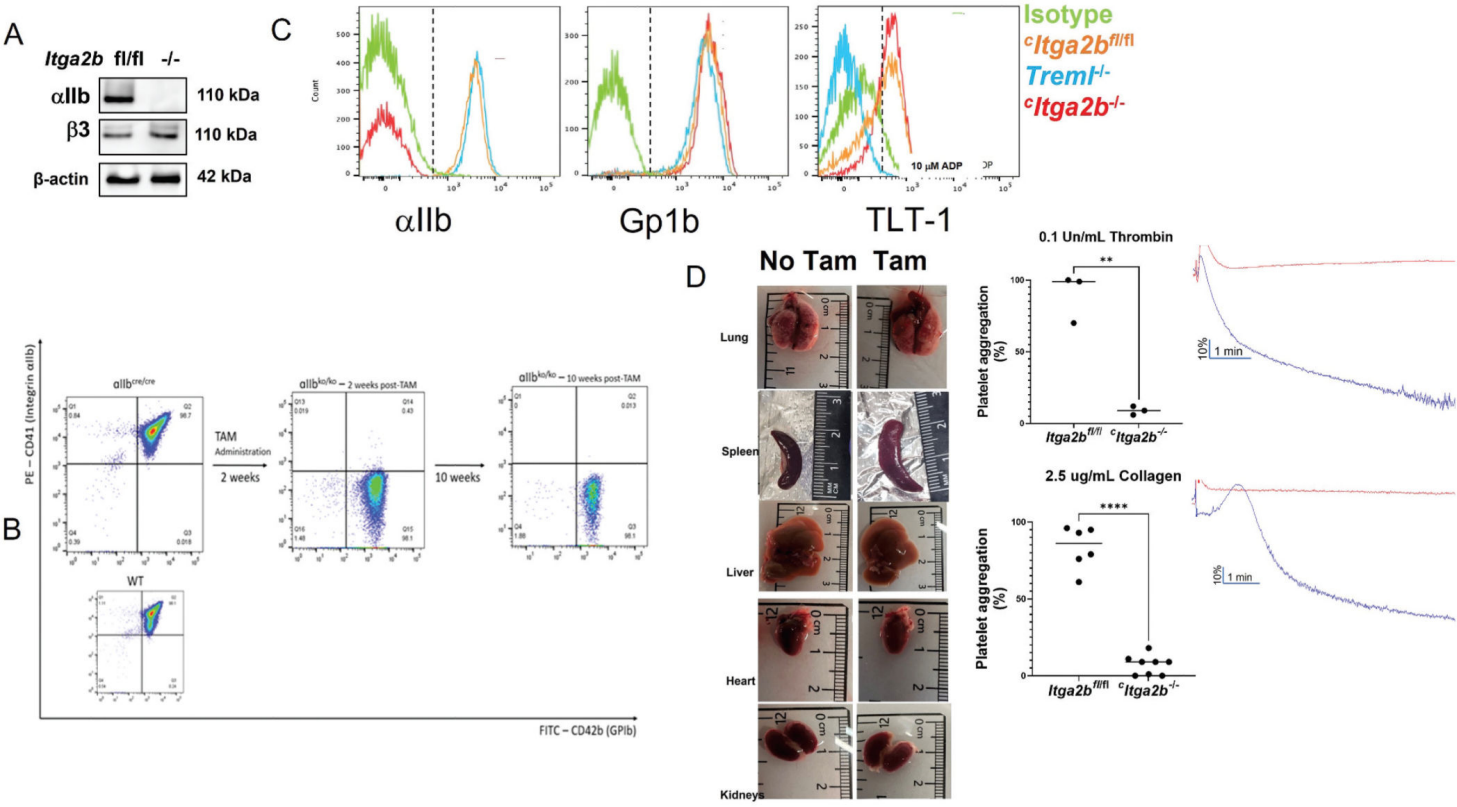
The ^*c*^*Itga2b*^*−/−*^ mouse has a Glanzmann thrombasthenia phenotype after tamoxifen diet. The presence or absence of αIIb was verified by A) Western blot and (B) flow cytometric analysis of ^*c*^*Itga2b* mice before (fl/fl) and after (^−/−^) 21 and 70 days of tamoxifen feeding. GPIb (x-axis) was used as an activation-independent platelet specific marker and CD41 PE to measure αIIb. Platelets were evaluated prior, immediately after, and 10 weeks post-tamoxifen diet. C) Flow cytometric analysis showing ^*c*^*Itga2b*^*−/−*^ mice have normal TLT-1 levels. D) ^*c*^*Itga2b*^*−/−*^ mice have splenomegaly after tamoxifen diet. The left side shows representative organs before tamoxifen diet; the right side is post-diet. E) ^*c*^*Itga2b*^−/−^ mice fail to aggregate after thrombin, or collagen activation. Quantification is shown on the left and analysis was by student T test, *n* = 3/group, Representative curves are shown on the right.

Descriptive results of continuous variables were expressed as mean ± SEM. A paired, two-tailed Student’s t-test analysis, conducted using Prism version 7.01 (GraphPad Software), was applied to evaluate statistical differences between the two groups (Figure 1D). Analyses of variance on ranks were performed using one-way ANOVA (analysis of variance) for more than two groups, followed by Bonferroni’s multiple comparison test. A p-value of < 0.05 was considered statistically significant. Clot retraction was analyzed by two-way ANOVA.

## Results

### Basic hemostatic characterization of the conditional itga2b^−/−^ (^C^itga2b^−/−^) mice

Floxed *itga2b* (*Itga2b*^*fl/fl*^) mice were born at normal Mendelian ratios and were fed tamoxifen. After two weeks, *Itga2b* gene expression was reduced to 6%, and by day 17, complete absence of expression was confirmed by PCR (Supplemental Figure S1), western blot (Figure 1A), and flow cytometry (Figure 1B, C). For further studies, mice were fed tamoxifen for 21 days and phenotyped by flow cytometry before use. While male mice tolerated the tamoxifen feeding, female mice were often undernourished, leading many to succumb to malnutrition and gastrointestinal bleeding. Since the mice experienced weight loss during the three weeks of tamoxifen feeding (Supplemental Figure S3A), an additional two weeks of normal chow feeding were required after tamoxifen treatment to restore the mice to a normal weight range. Although the ^*c*^*Itga2b*^*−/−*^ mice expressed *Itgb3* transcript, the Itgb3 protein (CD61) was not seen on the platelet surface (Supplemental Figure S2). The ^*c*^*Itga2b*^*−/−*^ mice showed normal levels of the receptors Gp1b, and TLT-1 (Figure 1C). After tamoxifen feeding, the conditional knockout mice recapitulated the Glanzmann’s thrombasthenia phenotype observed in global *Itga2b*^*−/−*^ mice^16^ and *Itgb3*^−/−^ mice.^25^ The mice exhibited enlarged spleens, significantly prolonged bleeding times compared to wild type (WT) and ^*c*^*Itga2b*^*fl/fl*^ mice and their platelets demonstrated an inability to aggregate in aggregation assays (Figures 1D,E and S4A). Consistent with these findings, ^*c*^*Itga2b*^−/−^ mice showed increased prothrombin time; however, their activated partial thromboplastin time was lower than that of *Itga2b*^*fl/fl*^ mice (Supplemental Figure S3B,C). Their factor V activity levels were not different from those of *Itga2b*^*fl/fl*^ littermate floxed controls (Supplemental Figure S3D). To test the effect of tamoxifen on platelet function, platelet aggregometry and spreading were conducted on platelets from mice that were fed chow or tamoxifen diet. No differences were seen between platelets from these mice (Supplemental Figure S4). These mice were then crossed with *Treml1*^*−/−*^ mice and evaluated for differences in hemostatic properties. *Treml1*^*−/−*^ platelets had not demonstratable differences on a tamoxifen and chow diets (Supplemental Figure S4). The characterization of these mice strains is shown in Supplemental Figure S1C–E.

### Basic hemostatic characterization of the conditional ^c^Itga2b^−/−^/Treml1^−/−^ (^c^DKO) mice

**Figure 2.**
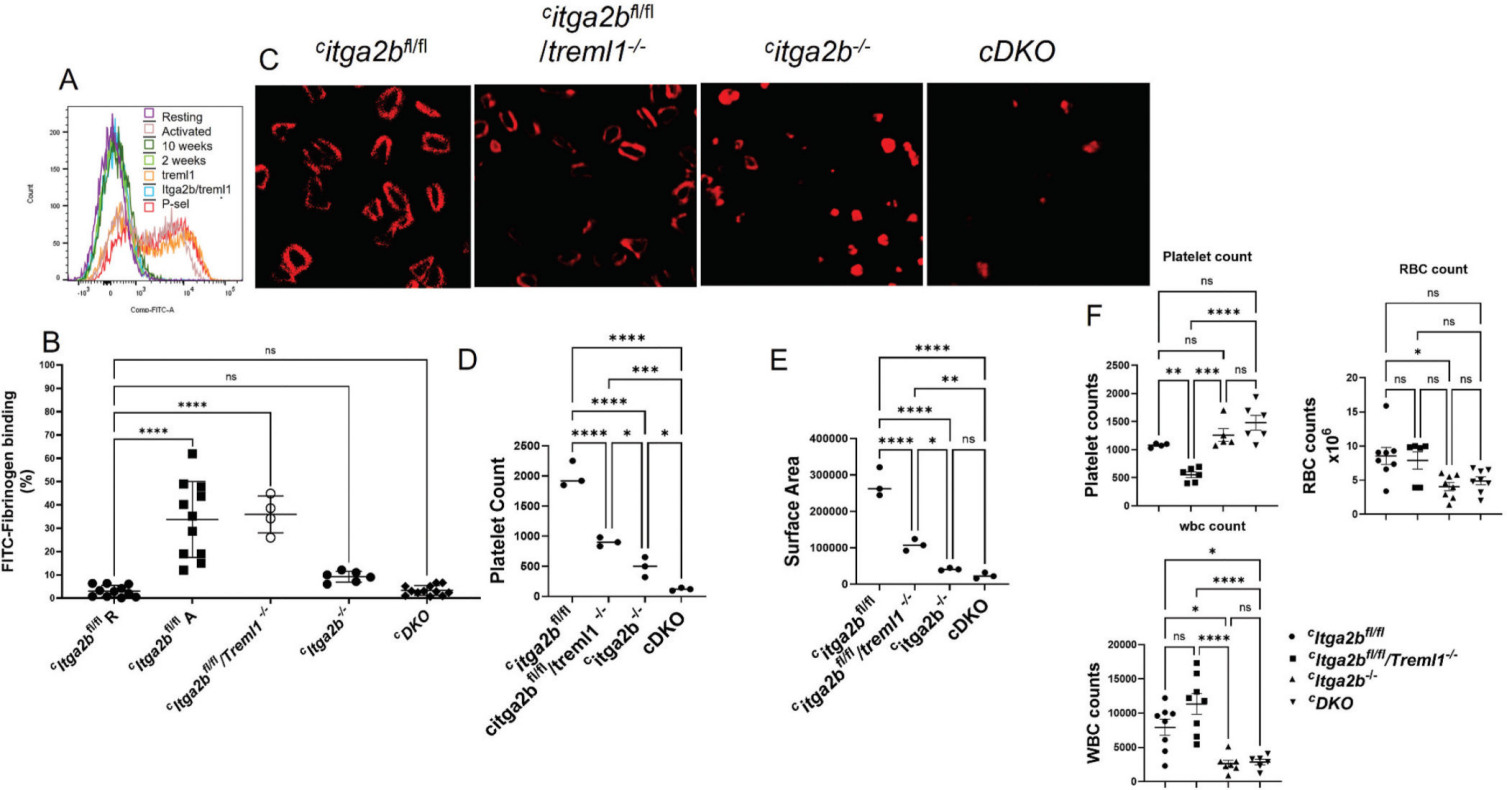
TLT-1 interaction with fibrinogen plays a supportive role in fibrinogen dependent platelet interactions. A, B) Representative flow cytometry curves of FITC labeled fibrinogen binding to washed platelets (A) and quantification is shown below (B), *n* = 4 – 11/group. Platelets were activated with 10 μM ADP unless designated with an R or resting in which no activator was added. (C) Platelets were spread on fibrinogen coated coverslips stained with rhodamine phalloidin; pictures were taken by confocal microscopy, *n* = 3/group. Representative pictures are shown, and quantification was completed using image J (D and E; *n* = 3). F) Blood cell counts measured by Sysmex hemocytometer. One-way ANOVA was used for statistical analysis, *n* = 4 – 8/group. (*) *p* < .05, (**) *p* < .01, (***) *p* < .001, (****) *p* < .0001

Since a key aspect of platelet function is based on αIIbβ3’s interaction with fibrinogen we used FITC labeled fibrinogen to measure platelet interaction in each strain of mouse. Measurement of FITC-labeled fibrinogen binding showed no significant differences between ^*c*^*Itga2b*^*fl/fl*^*/Treml1*^*−/−*^ *and* ^*c*^*Itga2b*^*fl/fl*^ strains. In contrast, ^*c*^*Itga2b*^−/−^ and ^*c*^*DKO* mice exhibited significantly reduced interaction with fibrinogen compared to both ^*c*^*Itga2b*^*fl/fl*^/*Treml1*^−/−^ and ^*c*^*Itga2b*^*fl/fl*^ strains, which were not significantly different from each other (Figure 2A,B). The platelet spreading assay was used to interrogate the receptors’ role in platelet spreading and adhesion (Figure 2C–E). The spreading assay revealed decreased adhesion and spreading area with all three mutant strains compared to ^*c*^*Itga2b*^*fl*/fl^ mice (*p=*0.0001); the ^*c*^*Itga2b*^*−/−*^ and ^*c*^*DKO* platelets failed to spread and had the lowest number of adherent cells and smallest area/platelet (Figure 2D). The average surface area of the ^c^DKO platelets was not significantly different than those of the ^*c*^*Itga2b*^*−/−*^ type (Figure 2E). In contrast, both ^*c*^*Itga2b*^−/−^ (1260 ± 254.1) and ^*c*^*DKO* (1480 ± 319.7) mice exhibited higher platelet counts, although neither was significantly different than those of the *Itga2b*^*fl/fl*^. However, both were significantly higher compared to ^*c*^*Itga2b*^*fl*/*fl*^/*Treml1*^*−/−*^.

**Figure 3.**
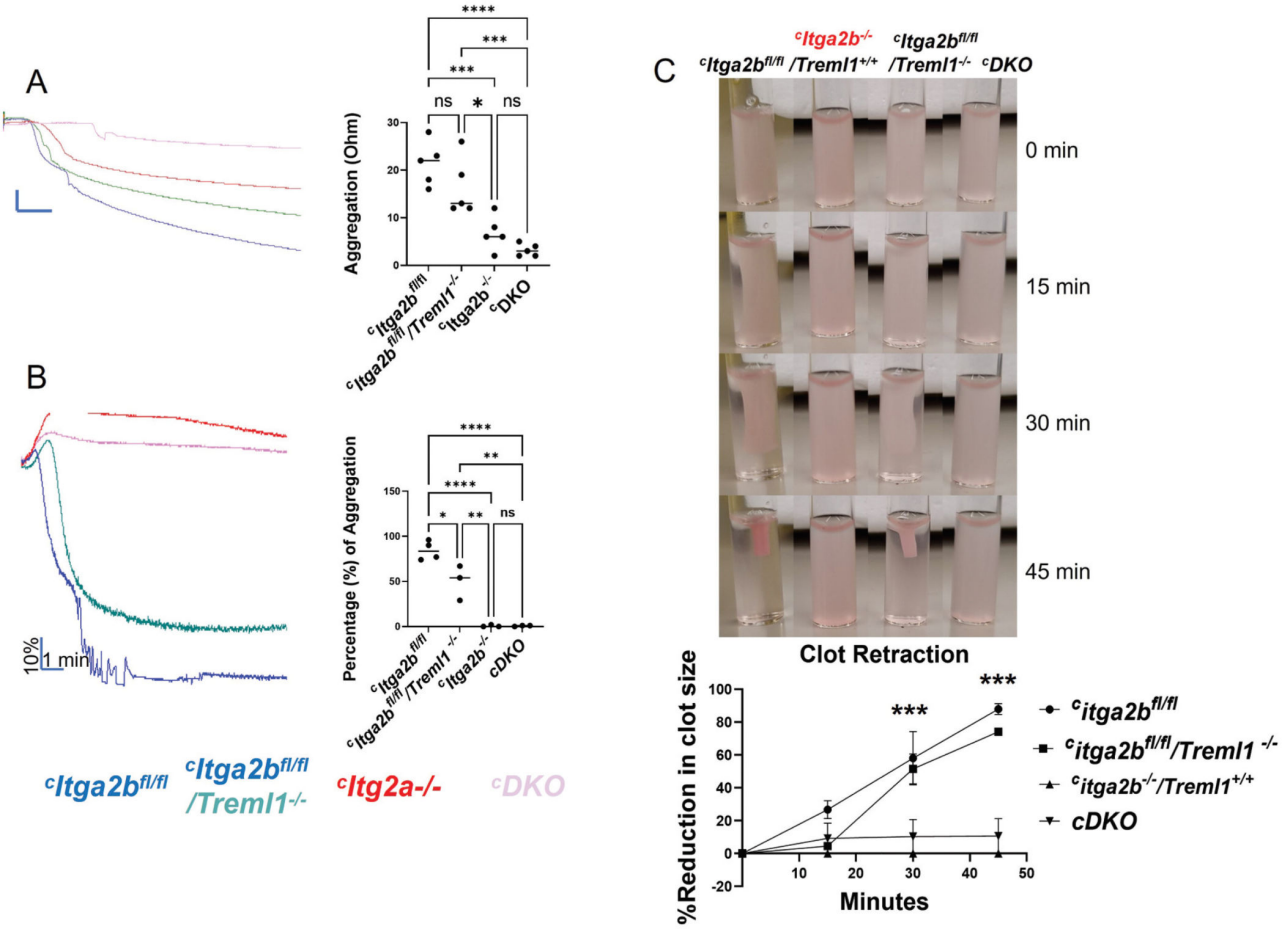
Evaluation of the role of αIIbβ3 and TLT-1 in platelet aggregation and clot retraction. A, B) Aggregation of platelets using (A) 0.1 un/mL thrombin and whole blood aggregometry top left and quantification (right, *n* = 5/group) (B) 5 μg/mL collagen using light transmission (bottom left) and quantification (right), one-way ANOVA was used for statistical analysis, *n* = 3–4 group. (C) Washed platelets activated with 1 un/mL thrombin was used for clot retraction. Representative image of clot retraction for the four strains (top) quantification (bottom) two-way ANOVA was used for statistical analysis, *n* = 3. (*) *p* < .05, (**) *p* < .01, (***) *p* < .001, (****) *p* < .0001

Platelet aggregation is central to clot formation, to measure this we used platelet aggregometry. Whole blood aggregometry with low-dose thrombin (0.1 U/mL) revealed significant differences between the parental *Itga2b*^*fl/fl*^ (21.4 ± 2.1 Ω) and ^*c*^*Itga2b*^*fl/fl*^/*Treml1*^*−/−*^ (16.4 ± 6.8) strains compared to the ^*c*^*Itga2b*^*−/−*^ (6.8 ± 1.6 Ω; *p* = .0003 and *p* = .01) and ^*c*^*DKO* strains (3.2 ± 0.5 Ω; *p* < .0001 and *p* = .009). Whole blood aggregation in ^*c*^*Itga2b*^*fl/fl*^*/Treml1*^*−/−*^ mice was not significantly different than *Itga2b*^*fl/fl*^ mice, and ^*c*^*Itga2b*^*−/−*^ was not different than ^*c*^*DKO* (Figure 3A). For platelet aggregation studies using collagen as the agonist, we used platelet-rich plasma and light transmission. The platelets of all strains showed a noticeably reduced aggregation response to collagen at 5 μg/mL compared to ^*c*^*Itga2b*^*fl/fl*^ controls (Figure 3B). ^*c*^*Itga2b*^*fl/fl*^ platelets aggregated at an average of 84.2 ± 5%, while ^*c*^*Itga2b*^*fl*/fl^/*Treml1*^*−/−*^ platelets aggregated at an average of 50 ± 11%. Both the ^*c*^*Itga2b*^−/−^ (0.6 ± 0.6%) and ^*c*^*DKO* (0.6 ± 0.3%) genotypes exhibited the greatest reduction in platelet aggregation. Platelets from the ^*c*^*DKO* mice were also unable to complete shape change. The difference between ^*c*^*Itga2b*^−/−^ and ^*c*^*DKO* was not significant.

Consistent with the aggregation results, αIIbβ3 played a critical role in the ability of platelets to mediate clot retraction. αIIbβ3 is critical to clot retraction.^26^ Figure 3C shows that both ^*c*^*Itga2b*^*−/−*^ and ^*c*^*DKO* mice exhibited minimal ability to retract clots, while ^*c*^*Itga2b*^*fl*/fl^/*Treml1*^*−/−*^ demonstrated a delay in retraction at the 15-minute time point.

**Figure 4.**
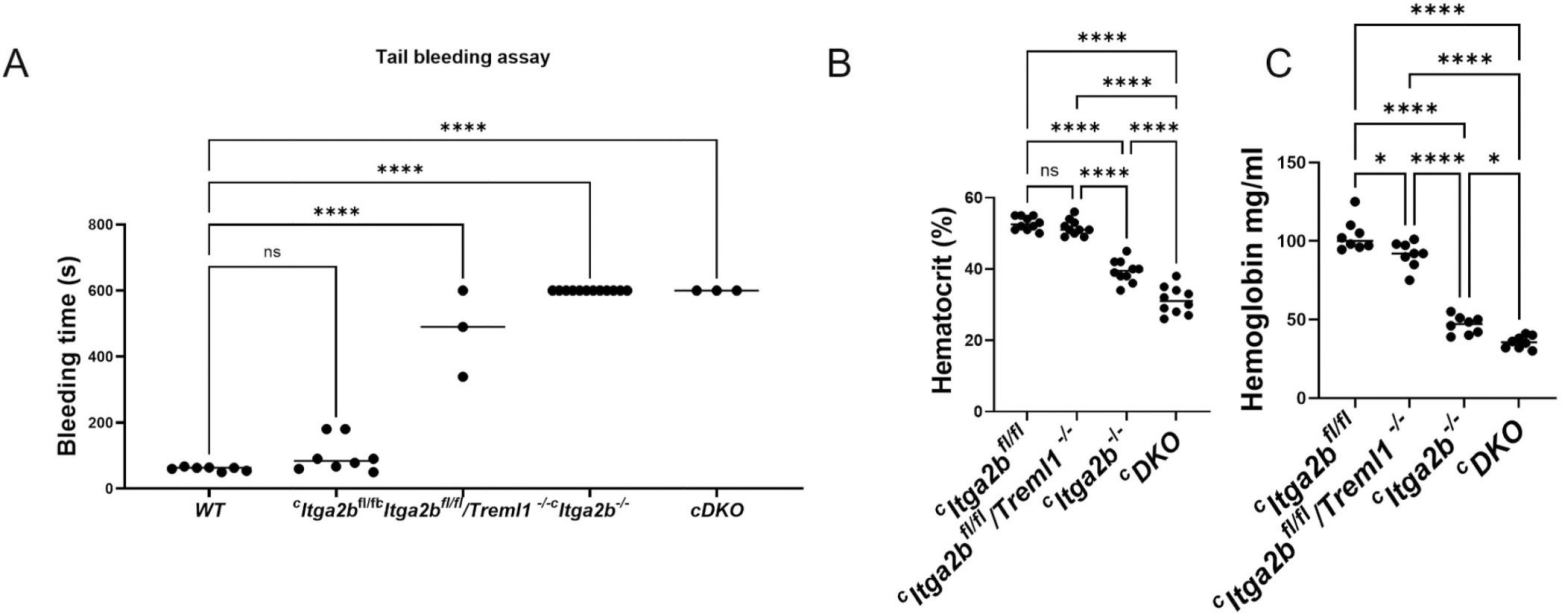
Physiological comparison of the ^c^DKO with parental strain’s basic hemostatic parameters. A) Bleeding time completed by tail clip assay as described in materials and methods, *n* = 3 −8/group. B) Hematocrit C) hemoglobin levels in DKO and parental strains of mice completed as described in material and methods. *n* = 7 and 11/group. One-way ANOVA was used for statistical analysis, (*) *p* < .05, (**) *p* < .01, (***) *p* < .001, (***) *p* < .0001

To understand the function of these αIIbβ3 and TLT-1 in hemostasis in vivo, we measured tail bleeding and the hemoglobin levels in the blood and hematocrit in the mutant and parental strains. Figure 4A shows the results of our tail bleeding assays, indicating that the difference in bleeding cessation between ^*c*^*Itga2b*^*−/−*^ and ^*c*^*DKO* mice was not significant. Wild type and ^*c*^*Itga2b*^*fl/fl*^ mice had tail bleeding times of 60 ± 2.3 seconds and 99 ± 18.3 seconds, respectively, while ^*c*^*Itga2b*^*fl/fl*^*/Treml1*^*−/−*^ mice exhibited prolonged bleeding times of 476 ± 75.7 seconds (*p* = .0001). As expected, ^*c*^*Itga2b*^−/−^ and ^*c*^*DKO* mice displayed significantly elevated bleeding times ( > 600 seconds) compared to parental strains. The mice required cauterization to stop the bleeding in this assay. The ^*c*^*Itga2b*^*fl*/fl^ and ^*c*^*Itga2b*^*fl/fl*^/*Treml1*^*−/−*^ mice had similar hematocrit (52.8 ± 1.0 vs 51.60 ± 2.2); however, the hemoglobin levels were much lower in the ^*c*^*DKO* mice (104 ± 3.6 vs 91.2 ± 2.0, *p* = .01; Figure 4B,C). The ^*c*^*Itga2b*^*−/−*^ (39.4 ± 1, 46.4) and ^*c*^*DKO* (31.0 ± 1.2) mice had significantly lower hematocrit (39.4 ± 1, 31 ± 1.2) and hemoglobin (46.4 ± 2.0, 35.5 ± 1.4) levels than ^*c*^*Itga2b*^*fl*/fl^ and ^*c*^*Itga2b*^*fl*/fl^/*Treml1*^*−/−*^ mice (*p* = .0001). The ^*c*^*DKO* mice also exhibited the most severe decreases in hematocrit and hemoglobin, showing significantly lower levels than any of the other strains, including ^*c*^*Itga2b*^*−/−*^ (*p* = .001 and *p* = .03, respectively).

### ^c^DKO platelets exhibited decreased fibrinogen deposition in the lungs and reduced platelet-neutrophil conjugate formation following inflammatory challenges

**Figure 5.**
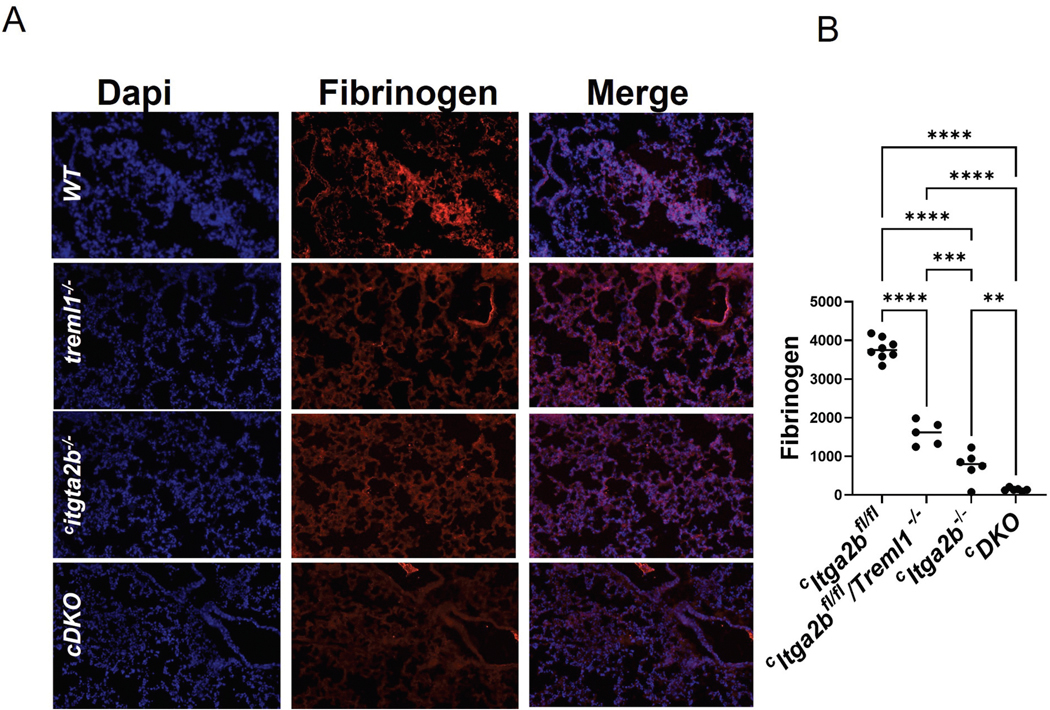
LPS affects fibrinogen deposition. A) Fibrinogen staining (red) DAPI (blue) staining of the lungs 24 hours after intranasal LPS treatment (B) quantification of immunofluorescent antibody staining completed in image J. One-way ANOVA was used for statistical analysis, *n* = 5 – 8/group. (*) *p* < .05, (**) *p* < .01, (***) *p* < .001, (****) *p* < .0001

Morales et al^22^ have previously demonstrated that patients with ARDS and elevated s-TLT-1 levels in a clinical setting were more prone to mortality, while TLT-1 regulated fibrinogen deposition in lungs after acute lung injury. To evaluate the immunological role of TLT-1 in our inducible model, we intranasally administered LPS to the mice and assessed fibrinogen deposition at 24 hours. Fibrinogen deposition in the lungs was decreased in ^*c*^*Itga2b*^*fl/fl*^/*Treml1*^*−/−*^, *Itga2b*^*−/−*^ and ^*c*^*DKO mice* when compared to ^*c*^*Itga2b*^*fl/fl*^ controls (Figure 5A,B). Although the differences between the ^*c*^*Itga2b*^*−/−*^ *and* ^*c*^*DKO* strains were small, they were statistically significant. There was no significant difference in platelet-neutrophil conjugate formation from bronchoalveolar lavage of the lungs (data not shown).

## Discussion

Both TLT-1 and αIIbβ3 bind fibrinogen; the latter, however, is the more abundant and prominent receptor on the platelet surface, which obscured our ability to objectively evaluate the role of each receptor. To address this issue, we developed a conditional *Itga2b* knockout model, enabling us to dissect the individual contributions of each receptor to hemostasis and inflammation.

The generation of a conditional *Itga2b*^−/−^ model was chosen to overcome the possibility that removal of two platelet fibrinogen receptors could be embryonically lethal. Our ^*c*^*Itga2b*^−/−^ recapitulated the Glanzmann thrombasthenia phenotype seen in humans and in the previously published *Itga2b* (αIIb) and *Itgb3* (β3) knockout mouse models.^25,27^ Genotypic analysis confirmed the successful platelet specific knockout of the *Itg2b* gene, which was validated by flow cytometric and Western blot analyses. Importantly, while β3 was expressed intracellularly, it failed to localize to the cell membrane in the absence of αIIb, confirming that αIIb is essential for the surface expression of the αIIbβ3 complex. Our ^*c*^*Itga2b*^−/−^ mice exhibited a severe bleeding diathesis, enlarged spleen and gastrointestinal bleeding, phenotypes similar to those reported in the global *Itga2b* knockout model. Interestingly, the prothrombin time was increased and the aPTT was decreased, this was surprising since clinically prothrombin time and aPTT are typically normal with only bleeding time changed and it is unusual to increased PT and decreased aPTT. Our findings could be a compensatory mechanism in these mice due to the acute, rapid decline in platelet numbers, unlike clinically in patients where Glanzmann Thrombasthenia is a chronic, lifelong condition. These findings may suggest an exhaustion of the contact pathway in our model and physiologically would warrant an evaluation of FVII. Feeding mice tamoxifen diets adversely affected the female mice leading to weight loss and even death in some instances. In retrospect, we would consider using a doxycycline-based Cre system to mitigate the tamoxifen toxicity observed in our female mice. Despite this challenge, we were able to evaluate the contributions of each receptor using this model.

In contrast to the severe bleeding phenotype observed in our ^*c*^*Itga2b*^*−/−*^ knockout mice, TLT-1 knockout mice exhibited a milder bleeding phenotype which may in part be due to the reduced platelet count. In the presence of αIIb, TLT-1 affected neither the fibrinogen binding to platelets nor hematocrit or hemoglobin levels. Interestingly, hemoglobin levels were reduced in αIIb knockout mice despite normal RBC counts, this may reflect an increased red blood cell turnover due to intestinal bleeding in the ^*c*^*Itga2b* null mice. Red and white blood cell counts were depressed in the ^*c*^*Itga2b*^−/−^ and ^*c*^*DKO* strains, but only the white blood cell counts were significantly different. Interestingly, the ^*c*^*Itga2b*^*fl/fl*^*/Treml1*^*−/−*^ mice exhibited decreased platelet counts and increased white blood cell counts, which were not significantly different than those of the ^*c*^*Itga2b*^*fl/fl*^ *mice*, but drastically different compared to the ^*c*^*Itga2b*^−/−^ and ^*c*^*DKO* strains. A notable variation was observed in platelet adhesion and bleeding time, but not in spreading, when compared to ^*c*^*Itga2b*^*fl/fl*^ mice. In the absence of αIIb, TLT-1 did not make a significant difference in fibrinogen binding to the platelets demonstrating that TLT-1 is not needed for fibrinogen adhesion to platelets. We were surprised to observe that in some instances (e.g., hematocrit, hemoglobin levels, and aggregation), the ^*c*^*DKO* exhibited a small yet notable change compared to the ^*c*^*Itga2b*^−/−^ mouse, revealing a mild yet significant supportive role for TLT-1 in the hemostatic functions of platelets. Because of the severity of the ^*c*^*Itga2b*^−/−^ phenotype, we did not use the FeCl_2_ assay to differentiate ^*c*^*Itga2b*^−/−^ and ^*c*^*DKO’s* ability to form intravascular thrombi. Other than the aggregation assay, there is a larger variation between the ^*c*^*Itga2b*^*fl/fl*^ and ^*c*^*Itga2b*^*fl/fl*^*/Treml1*^*−/−*^ mice than the ^*c*^*Itga2b*^*−/−*^ and the ^*c*^*DKO*. These data reveal that TLT-1’s function may not be adhesive in nature. Nonetheless, the notable differences observed in hematocrit, hemoglobin levels, and aggregation support a significant role for TLT-1 in hemostasis.

The TLT-1 bleeding diathesis has always been the most apparent after LPS challenge.^19,22^ In this study, we challenged mice intranasally with LPS and evaluated fibrinogen deposition in the lungs. Consistent with the hemostatic results, there was a marked drop in fibrinogen deposition between the ^*c*^*Itga2b*^*fl/fl*^ and ^*c*^*Itga2b*^*fl/fl*^*/Treml1*^*−/−*^ strains. The ^*c*^*DKO* mice exhibited a small yet significant reduction in lung fibrinogen deposition, supporting the notion that the TLT-1/fibrinogen interaction is not a primary adhesive force. In our previous studies, higher levels of fibrinogen deposition were associated with reduced alveolar damage.^22^

Taken together, our findings demonstrate that TLT-1 does not support clot formation through adhesive binding to fibrinogen. The lack of a significant difference in fibrinogen binding between ^*c*^*Itga2b*^*−*^*/*^*−*^ and cDKO strains confirms that TLT-1 does not participate in platelet cohesion. These results establish that the role of the TLT-1/fibrinogen interaction lies outside of adhesion, making it essential to define alternative functions of this pathway in thrombus regulation. In our previous work, we showed that MIP-2 stimulation of the cremaster muscle induces microvascular clot formation in wild-type mice, whereas *Treml1*^*−*^*/*^*−*^ mice exhibit significantly reduced microclot formation.^28^ Placed in this context, the present study indicates that the TLT-1–fibrinogen interaction is not adhesive but instead supports the stabilization of nascent clots.^29^

## Supplementary Material

Supp 1

## Plain Language Summary

### What is the context?

- The study focuses on the role of platelets, specifically the fibrinogen-binding receptors αIIbβ3 and TLT-1, in clot formation, immune response, and inflammation.
- The αIIbβ3 integrin is a well-established receptor for platelet aggregation, essential for blood clotting, while TLT-1, although known to interact with fibrinogen, has unclear functions.
- TLT-1’s interaction with fibrinogen is thought to participate in platelet-platelet adhesion.
- Researchers are interested in understanding how these receptors contribute to both hemostasis (preventing bleeding) and inflammation, especially in conditions like atherosclerosis, and sepsis, where platelet dysfunction plays a role in disease progression.

### What is new?

- The study uses a genetic approach to develop a conditional knockout model targeting αIIbβ3 specifically in platelets, which mimics a bleeding disorder called, Glanzmann’s thrombasthenia.
- By crossing these knockout mice with TLT-1 knockout mice, researchers created a conditional double knockout (^c^DKO) to compare the contributions of both receptors in clotting and inflammation.
- The ^c^DKO model demonstrated that the changes platelet aggregation, bleeding times, and fibrinogen deposition in the lungs post-inflammation were significant between control mice and the single αIIbβ3 knockout mouse, but very small between the αIIbβ3 knockout mouse and the ^c^DKO.
- The data reveal thatthe TLT-1–fibrinogen bond is not responsible for platelet cohesion.

### What is the impact?

- The ^*c*^*Itga2b*^*−/−*^ model allows for selective removal of CD41/CD61 integrin from the platelet surface for other platelet studies.
- This pivotal finding changes the course of our investigation to explore novel, non-adhesive functions of this interaction.
