# Supplementary material for: Use of a conditional Glanzmann thrombasthenia mouse model reveals a supportive and possibly non-adhesive role for TLT-1 in the platelet-fibrinogen interaction
Source: Platelets. Author manuscript; Available in PMC 2026 Jan 15. (PMC12805433; doi:10.1080/09537104.2025.2574379)
Supplement: Supp 1 [file NIHMS2122171-supplement-Supp_1.docx]

**Use of a Conditional Glanzmann Thrombasthenia Mouse Model Reveals a**

**Supportive and Possibly Non-adhesive Role for the TLT-1 in the Platelet-**

**Fibrinogen Interaction**

**Supplementary figures**

Branfield, S.L., Koshy, N.P. Somani, Y., Manfredi, B., Schneider, C.D, Westrick, R.J^1^, and €Washington,

A.V.

Oakland University Department of Biological Sciences, Rochester MI, 48309

1Department of Bioengineering, Institute for Data Science, Oakland University, Rochester, MI 48309, and Life Sciences Institute, University of Michigan, Ann Arbor, MI 48109

Supplemental Figure 1


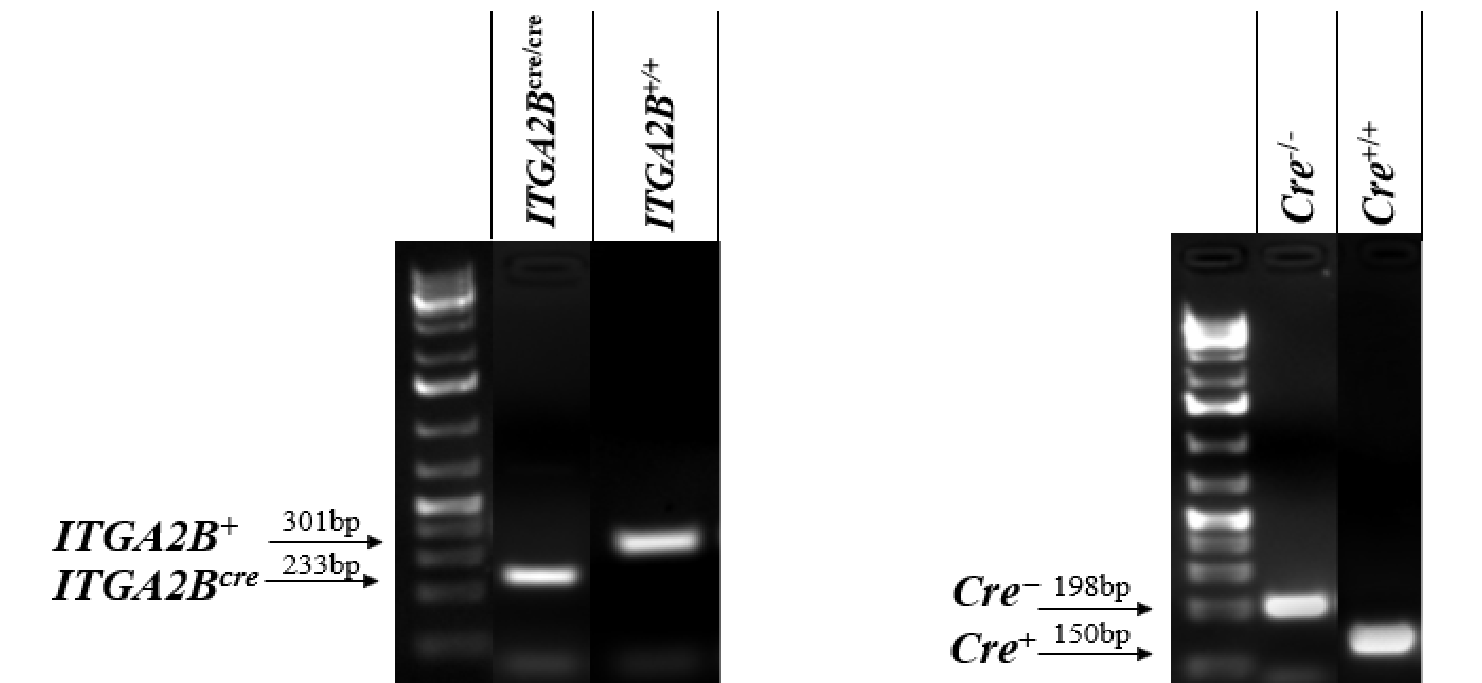
A
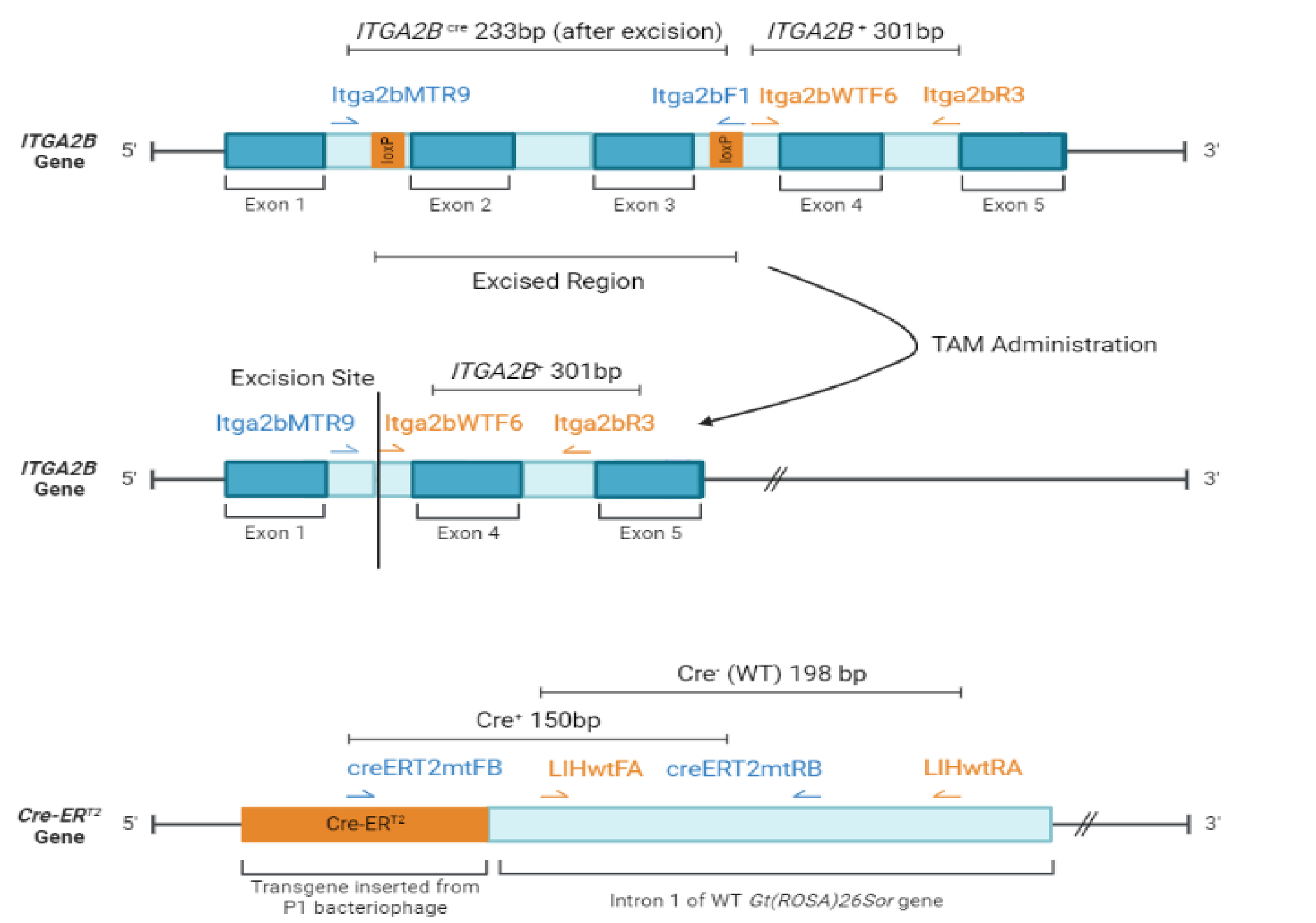
 B

#
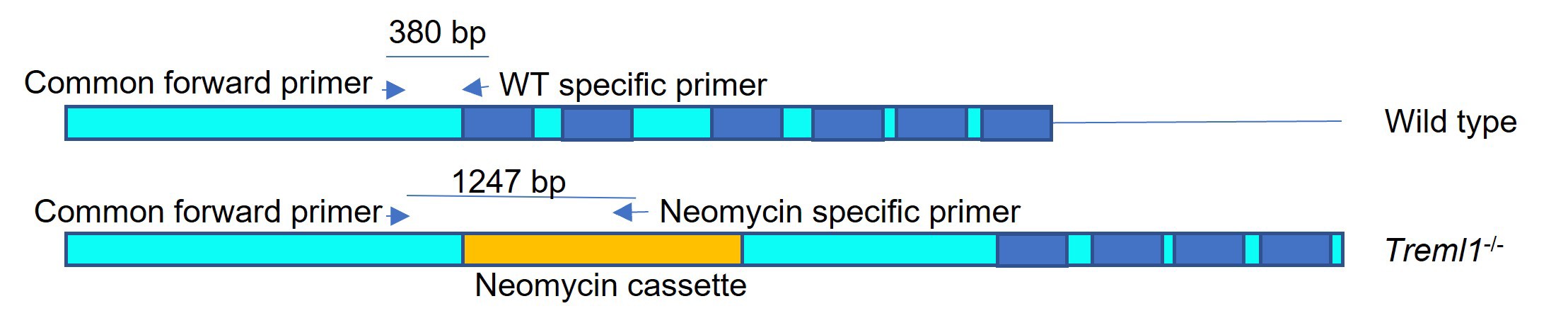
D
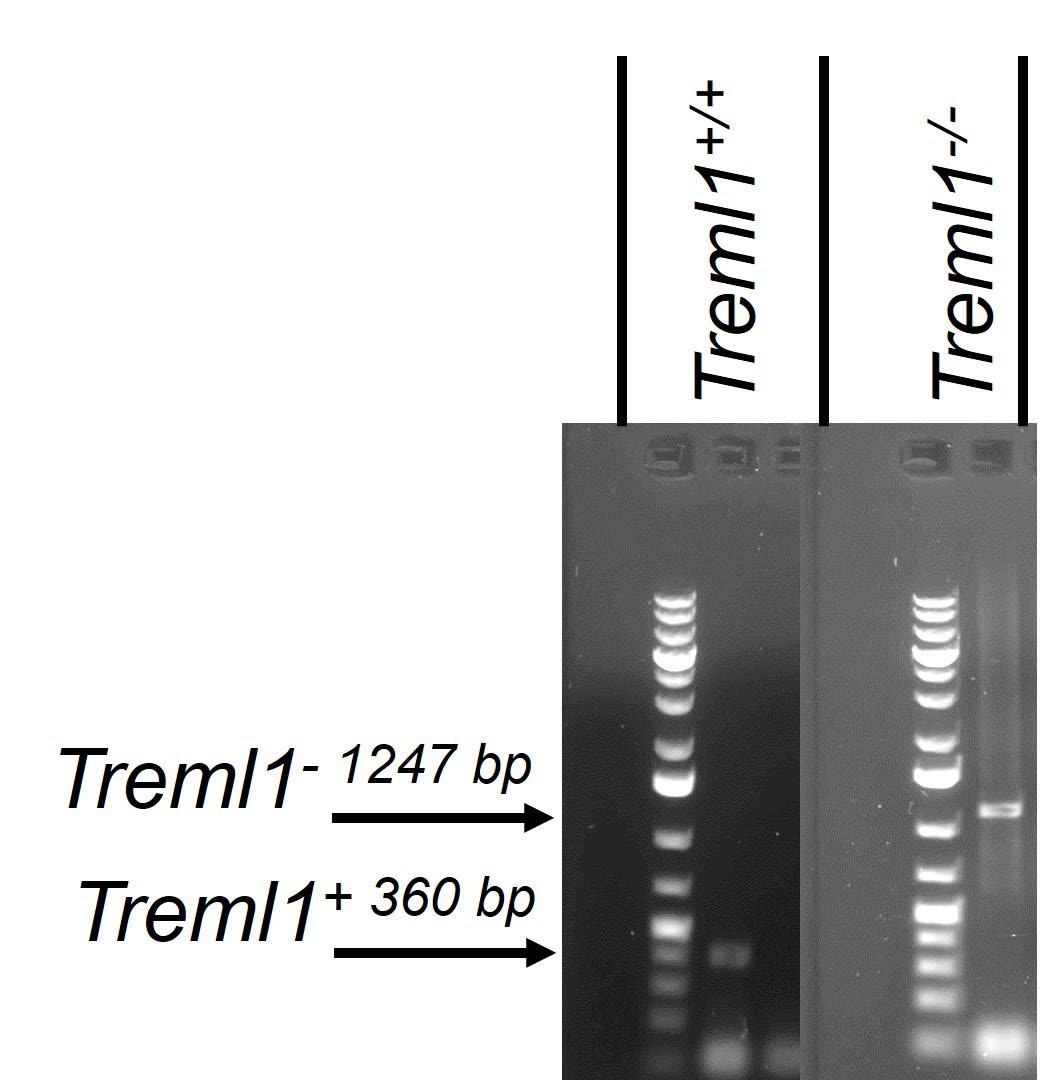


## **Generation of *^c^itga2b^-^*^/-^ Treml1-/-, and DKO Mice – Genotyping and Western blots**

We collaborated with Jackson Laboratories (JAX, Bar Harbor, ME) to create mice with loxP sites flanking exons two and three of the *itga2b* gene and obtained mice carrying the conditional Cre-ERT2 gene cassette of strain Gt(ROSA)26Sortm1(cre/ESR1)Tyj. These mice were intercrossed to produce offspring homozygous for both mutant alleles (*itga2b^fl^*^/fl^ and *Cre*^+/+^), which are responsive to tamoxifen treatment. Following tamoxifen administration, the inhibition of Cre-recombinase by the ERT2 moiety is lifted, leading to the expression of active Cre-recombinase. This results in the excision of exons two and three from the itga2b allele, causing a frameshift mutation in exon four and introducing a premature stop codon at codon 85. This alteration is expected to trigger nonsense-mediated decay of the transcript, converting the *itga2b* gene from a + to - state and significantly reducing or entirely eliminating the expression of integrin αIIbβ3 on platelet surfaces. Conventional polymerase chain reaction (PCR) was employed to genotype DNA extracted from tail biopsies of the offspring to confirm the excision.

Four reactions were carried out to amplify floxed *itga2b* and the transgenic, Cre-positive (Cre+) and wildtype, Cre-negative (Cre-) DNA. The following combinations of primers were used: for wildtype ITGA2B, forward primer Itga2bWTF6 (5’ AAT CCA GGG TAG GGA CAC 3’) and reverse primer Itga2bR3 (5’ TGG CAA ACC AGA GCT TAC GA 3’); for floxed ITGA2B, forward primer Itga2bF1 (5’ TAG GCT CCT CAA CCC TGT CA 3’) and reverse primer Itga2bMTR9 (5’ CGA AGT TAT TGG TTT GGT 3’); for transgenic Cre, forward primer creERT2mtFB/olMR3621 (5’ CGT GAT CTG CAA CTC CAG TC 3’) and reverse primer creERT2mtRB/olMR9074 (5’ AGG CAA ATT TTG GTG TAC GG 3’); for wildtype Crenegative, forward primer LIHwtFA/21306 (5’ CTG GCT TCT GAG GAC CG 3’) and reverse primer LIHwtRA/olMR9021 (5’ CCG AAA ATC TGT GGG AAG TC 3’). Predicted primer alignments and PCR results are shown.

For PCR, we set the final primer concentration at 0.5 µM, using NovaTaq Polymerase master mix (Millipore Sigma) and the T100 thermal cycler (Bio-Rad). The PCR conditions were as follows: melting at 94°C for 30 seconds; annealing at 50°C for floxed *itga2b*, 55°C for transgenic Cre, and 56°C for both wildtype reactions, each for 30 seconds; and extension at 72°C for 45 seconds for a total of 34 cycles. The amplified samples were resolved on a 2% agarose gel alongside a 1Kb Plus DNA ladder (Invitrogen) to estimate the length of the amplified DNA. This genotyping method was routinely employed before proceeding with subsequent experiments. Mice carrying the Itga2b mutation and cre incertions were bred with Treml1^-/-^ to create the Itga2b^fl/fl^ and DKO strains.

A) Schematic representing the *itga2b* and *cre* loci. B) Genotyping performed prior to tamoxifen administration should produce a 233bp product shown *as itga2b^-/-^*, a 301bp product for wildtype *itga2b*+ (left panel), a 150bp product for mutant Cre+, and a 198bp product for wildtype Cre- mice (right panel). C and D) Three-primer PCR was carried out for genotyping tail DNA from offspring. Common forward primer 1 (5’GGGGTACCTTGAGAATCAGATGGCCCTG-3′) was located at 5′ of the pgk-neo cassette; reverse primer 2 (5′-

CGGCACATGTGGCAGCTCGTCCATGCCGAGAGTG-3′) was neo cassette specific, and reverse primer 3 (5′-AGCAGCAGGTAGCAGTCCATG3′) was WT specific. The PCR products were 1247 base pairs (mutant) and 360 base pairs (WT) .

## Supplemental Figure 1E


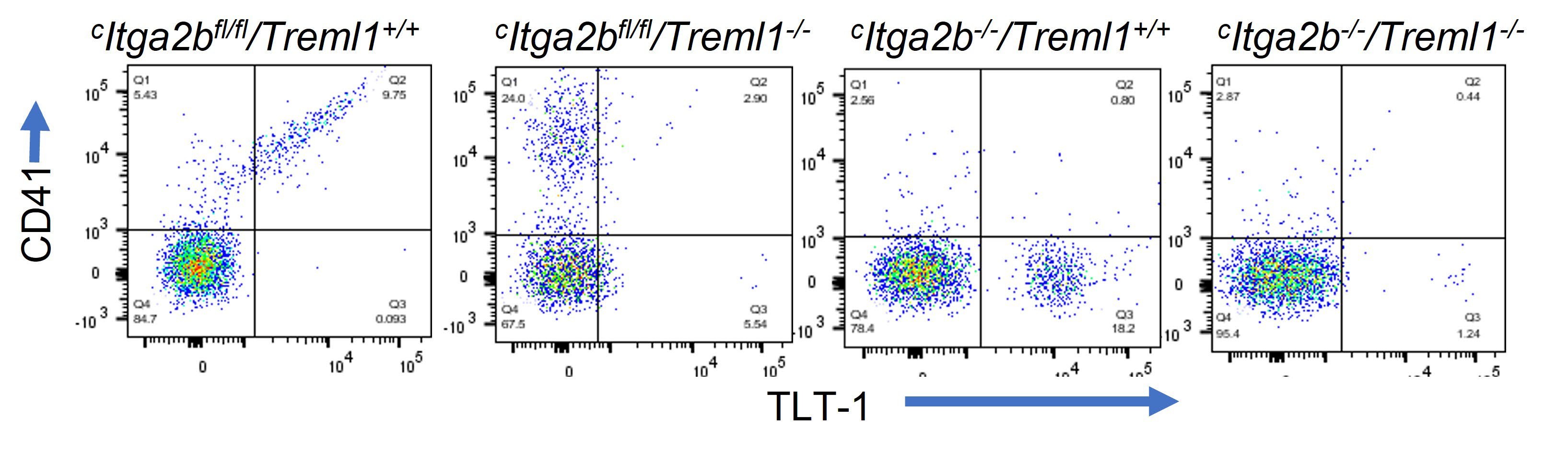

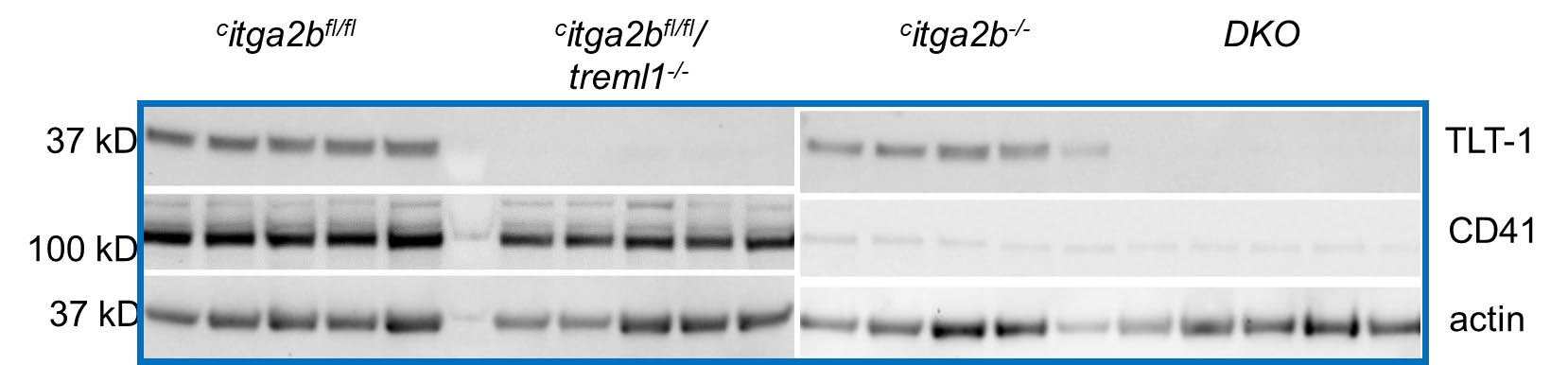


Characterization of the mutant strains by western blot and flow cytometry. Top) Absence of the TLT-1 and CD41 proteins was determined by Western blot and confocal analysis using rabbit monoclonal antibodies developed by this laboratory and anti CD41. Figure shows a of platelets activated with thrombin and lysed with 1% triton X lysis buffer according to previously published protocols (Manfredi *et al.*, doi:

10.1089/mab.2017.0063). Platelets from three to six mice were used for each lane. Blots were cut at the 50 KD marker and probed individually, The TLT-1 blots were stripped and re-probed with actin as a loading control. Bottom) Whole blood flow cytometry using 5 uL of blood retrieved by reto-orbital bleed and incubated with antibodies in Tyrode’s buffer with 10 µM ADP according to reference 19. Blood was incubated with antibodies for 15 minutes, fixed and read on a BD FACS Canto instrument. Figures were made using Flow Jo (FlowJo v10).

## Supplemental Figure 2
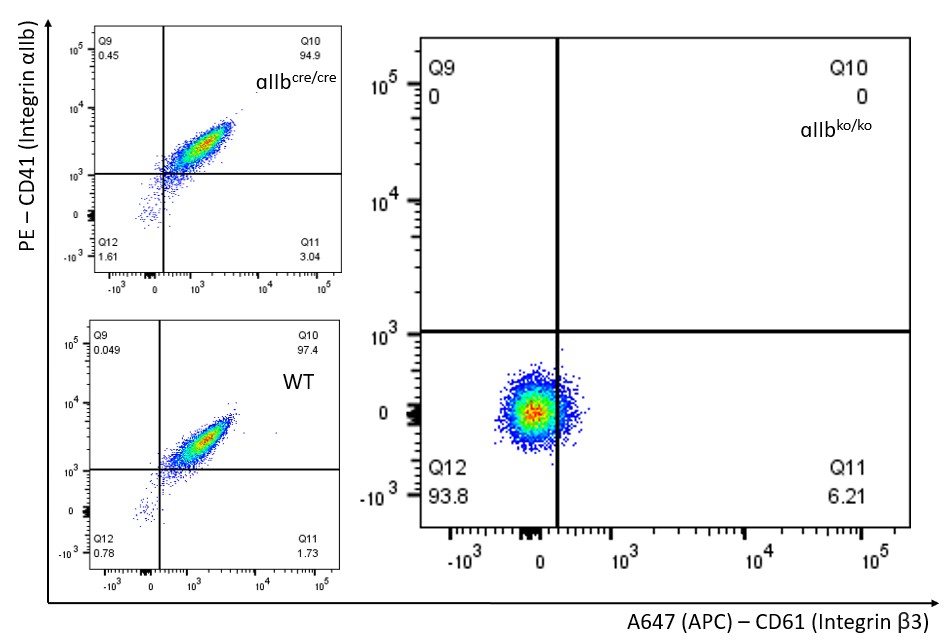


**Tamoxifen Administration Eliminates Expression of CD61 on cKO Platelets Through 10 Weeks.** This figure depicts results of the same mice at 10-weeks post-TAM administration as previously described, Tamoxifen also causes the elimination of CD61 cell surface expression (x-axis) These mice exhibit a CD41-/CD61- null phenotype which is maintained for a minimum of 10 weeks

✱✱✱

## A B Figure 3
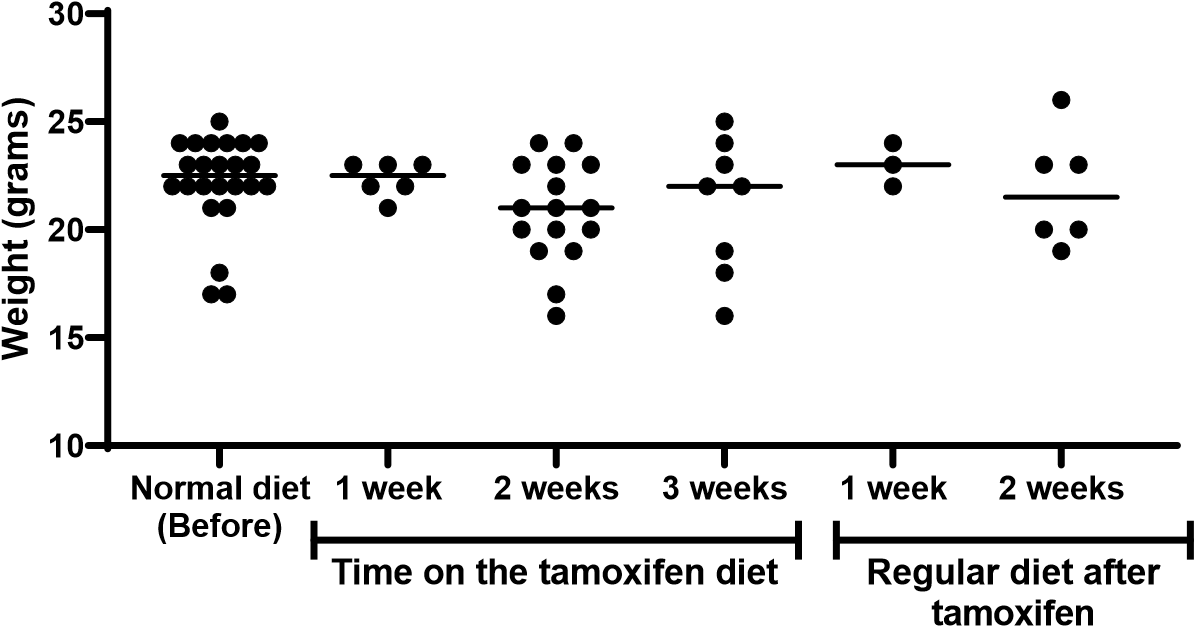


***c***

***itga2b***

**fl/fl**

***c***

***itga2b***

***/***

***-***

***-***

**7.0**

**7.5**

**8.0**

**8.5**

**9.0**

**9.5**

**Mouse Strain**

**Prothrombin time**

**seconds**

**)**

**(**

Supplemental **_Weight variability_**

✱✱ ns

**c**

**fl/fl**

***c***

***-***

**/-**

**25**

**30**

**35**

**40**

**Activated partial**

**thromboplastin time**

**(**

**)**

**seconds**

**0**

**20**

**40**

**60**

**80**

**100**

**Factor V**

**(**

**%**

**)**

C

D

**itga2b *Itga2b citga2b*fl/fl *citga2b-/-***

**Mouse Strain**

### Mouse Strain

**Mouse Weight Change and Coagulation Parameters in citga2b mice**. Mice with a conditional knockout of the integrin alpha 2b (itga2b) gene under the regulation of a tamoxifen-inducible Cre-recombinase (CreER/T2) transgenic mice were fed with 40 mg/kg tamoxifen. (A) Average mouse weights dropped after two weeks on tamoxifen diet. The increase seen at three weeks was due to the mice with lower weights succumb to intestinal bleeding. (B) Prothrombin time was significantly higher in *itga2b^-/-^* mice and (B), activated partial thromboplastin time was significantly lower p<0.01 (C., (D) There was no difference in Factor V activity percentage

***Thrombin Collagen***


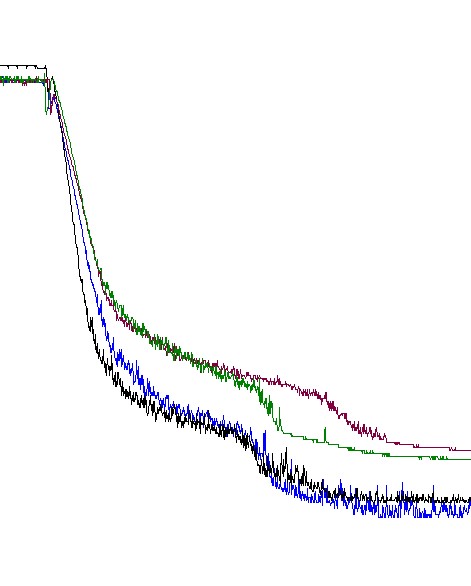

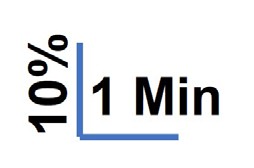

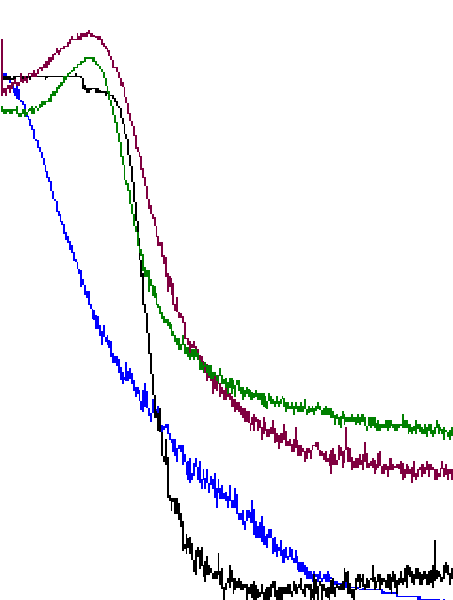

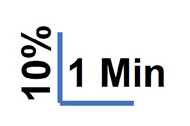


Supplemental **Platelet Spreading**

Figure 4^✱^

## ***WT, WT* Tamoxifen, *Treml1^-/-^,Treml1^-/-^* Tamoxifen**

**WT**

**WT Tamoxifen**

***Treml1***

***-***

***/-***

***Treml1***

***-***

***-***

***/***

**Tamoxifen**

**0**

**1000**

**2000**

**3000**

**4000**

**Genotype**

**Platelet counts**

ns

ns

✱

✱✱

**Tamoxifen Administration does not have significant effect on platelet functions.** Wild Type and *Treml1^-/-^* mice were fed tamoxifen or chow diet for three weeks and then allowed at least two weeks before harvesting platelets for aggregometry experiments and platelet spreading assays. For platelet aggregation, the curves are representative of three individual experiments. In the platelet spreading assays, the number of platelets adhered were counted in 7 fields/group. One-way ANOVA was used for statistical analysis, n = 4 – 6/group
